# JOSEPH T. FREEMAN AND EXCELLENCE IN REHABILITATION OF AGING PERSONS AWARD LECTURES

**DOI:** 10.1093/geroni/igz038.1301

**Published:** 2019-11-08

**Authors:** Christine Mueller

**Affiliations:** University of Minnesota, Minneapolis, Minnesota, United States

## Abstract

The Joseph T. Freeman Award is a lectureship in geriatrics awarded to a prominent physician in the field of aging, both in research and practice. The award was established in 1977 through a bequest from a patient’s estate as a tribute to Dr. Joseph T. Freeman. The Joseph T. Freeman Award lecture will feature an address by the 2019 Freeman Award recipient, Anne Newman, MD, MPH, of the University of Pittsburgh. The Excellence in Rehabilitation of Aging Persons Award is designed to acknowledge outstanding contributions in the field of the rehabilitation of aging individuals. The Excellence in Rehabilitation of Aging Persons Award lecture will feature an address by the 2019 Excellence in Rehabilitation Award recipient, Neil Alexander, MD, of the University of Michigan.

